# Associations between dietary antioxidant intakes and cardiovascular disease

**DOI:** 10.1038/s41598-022-05632-x

**Published:** 2022-01-27

**Authors:** Parvin Mirmiran, Firoozeh Hosseini-Esfahani, Zohreh Esfandiar, Somayeh Hosseinpour-Niazi, Fereidoun Azizi

**Affiliations:** 1 Nutrition and Endocrine Research Center, Research Institute for Endocrine Sciences, Shahid Beheshti University of Medical Sciences, Tehran, Iran; 2 Endocrine Research Center, Research Institute for Endocrine Sciences, Shahid Beheshti University of Medical Sciences, Tehran, Iran

**Keywords:** Diseases, Medical research

## Abstract

Cardiovascular disease (CVD), as the leading cause of death worldwide, is the collective term named for disorders afflicting the blood vessels and heart. Inflammation and enhanced oxidative stress have been shown as fundamental risk factors in the onset and progression of CVD. Chronic inflammatory conditions attenuate blood levels of antioxidants because of the continuous generation of elevated levels of reactive oxygen species (ROS). A sufficient intake of antioxidants is also suggested to beneficially interfere with CVD by quenching ROS. Antioxidant vitamins and minerals, such as vitamins A, E, and C, and zinc may slow the development and progression of CVD. This study aimed at investigating the association between daily consumption of dietary vitamins A, E, and C, and zinc and the incidence of CVD. Eligible adults (n = 5102) aged ≥ 30 years, were selected from the participants of the Tehran lipid and glucose study with an average follow-up of 5.3 years. Dietary intake was assessed using a valid and reliable semi-quantitative food frequency questionnaire. Anthropometrics and biochemical variables were evaluated at baseline and follow-up examinations. Multivariable Cox proportional hazard regression models were used to estimate the development of CVD associated with total intakes of vitamins A, E, and C, and zinc. This study was conducted on 2253 men and 2849 women aged 47.0 ± 11.6 and 45.6 ± 10.5 years, respectively. The main sources of dietary vitamins A, E, and C and zinc were fruits, vegetables, and legumes. Risk of CVD decreased from quartile 1 to quartile 4 for vitamin E intake (HR 1.00, 0.91, 0.77, and 0.57; *P*_*trend*_ = 0.03). The association between the risk of CVD and quartiles of vitamins A, and C and zinc intake was not statistically significant. Our study suggests an inverse association between vitamin E intake and the risk of CVD, emphasizing the potential protective role of fruit and vegetable in the prevention of CVD.

## Introduction

Cardiovascular disease (CVD), as the leading cause of death worldwide, is the collective term named for disorders afflicting the blood vessels and heart that accounts for 17.9 million deaths in 2016^1^. Inflammation and enhanced oxidative stress have been shown as fundamental risk factors for the onset and progression of CVD^2^. Chronic inflammatory conditions attenuate blood levels of antioxidants because of the continuous generation of elevated levels of reactive oxygen species (ROS). Sufficient intake of antioxidants is also suggested to beneficially interfere with CVD by quenching ROS^3^.

Antioxidant vitamins and minerals, such as vitamins A, E, and C, and zinc may slow the development and progression of CVD^4,5^. Observational epidemiological studies have suggested that a higher circulating concentration of vitamin E was associated with a lower risk of CVD^6^. Therefore, these findings indicate the need for additional and more accurate clinical investigations in this area.

Vitamin C is a major water-soluble antioxidant in plasma, and observational studies have shown an inverse association between dietary vitamin C and CVD outcomes^7,8^. However, in several large randomized controlled trials, the effect of vitamin C to prevent CVD has not been confirmed. Although the antioxidant potential of vitamin A was first determined in 1932^9^, limited data are available regarding the association between vitamin A and the risk of CVD^10^. Zinc is an essential trace metal with antioxidant and anti-inflammatory activities; insufficient intake of zinc has persistently been reported in CVD patients^11^. Trace element analysis of hair showed that patients with CVD had lower levels of zinc^12^. A 6-year follow-up cohort study on CVDs and dietary zinc intake reported no association between CVD and total zinc intake^13^; therefore, further investigations, as well as additional evidence obtained by observational studies are required.

Accordingly, studies on the association between CVD and the antioxidant vitamins and minerals such as vitamins A, E, and C and zinc cannot shape the dietary guidelines regarding the nutritional value of these factors^13,14^; therefore, the need to investigate the risk of CVD by dietary antioxidant vitamin and mineral intakes is warranted. Also, as far as we know, there is limited evidence about the association between antioxidant vitamins or minerals and the risk of CVD in Asian populations^14^. Hence, we aimed to prospectively evaluate the association between dietary antioxidant intakes (vitamins A, E, and C and zinc) and the risk of CVD in a group of adults in Tehran, Iran.

## Methods

### Study population

In this cohort study, the subjects were selected from participants of the Tehran lipid and glucose study (TLGS), a population-based prospective study to determine the risk factors for non-communicable diseases among the residents of District 13, Tehran, the capital city of Iran^15,16^. The first examination survey was performed from 1999 to 2001 on 15,005 individuals aged ≥ 3 years, using the multistage stratified cluster random sampling, and follow-up examinations were conducted every 3 years; 2002–2005 (survey 2), 2005–2008 (survey 3), 2008–2011 (survey 4), and 2012–2015 (survey 5) to identify recently developed diseases.

**Figure 1:**
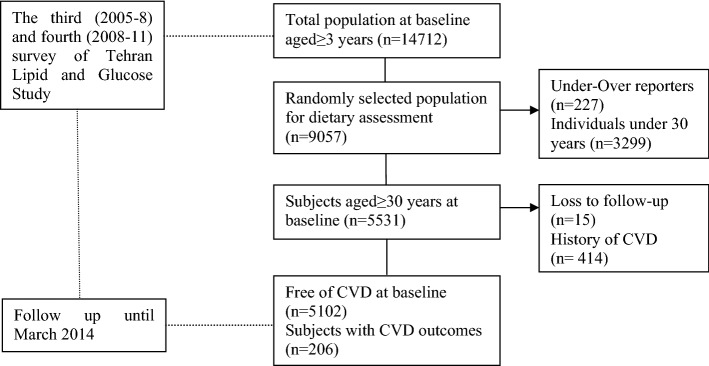
Outline of study participants’ selection.

Of 14,712 individuals participating at baseline (surveys 3 and 4), 9057 subjects were randomly selected for dietary assessment based on age- and sex-stratified random sampling, of whom 5531 subjects aged ≥ 30 years at baseline with completed data were included and followed until 2014. Subjects (n = 227) with under- or over-reporting of energy intake (< 800 or ≥ 4200 kcal/day)^17^ and also those with a history of CVD (n = 414) at baseline were excluded. Finally, after excluding participants with missing any follow-up data (n = 15), 5102 subjects remained and entered in the analysis (Fig. 1).

All participants signed a written informed consent form before the research. The study was conducted based on the Declaration of Helsinki and the study protocol was accepted by the Ethics Committee of the Research Institute for Endocrine Sciences, Shahid Beheshti University of Medical Sciences, Tehran, Iran. All methods were performed in line with the relevant guidelines and regulations. Materials and methods were checked using the strengthening of the reporting of observational studies in epidemiology (STROBE) statement.

### Dietary intake measurements

Dietary assessment was performed by a valid and reliable 168-item semi-quantitative food frequency questionnaire (FFQ)^18,19^. Trained interviewers collected information on usual dietary intake through face-to-face private interviews. The frequency of eating each food item on a daily, weekly, or monthly basis was recorded and daily intakes was computed via the consumption frequency multiplied by the portion size of each food item, and then the portion sizes were converted into grams using household measures. The weight of seasonal foods, like some fruits, was calculated based on the number of seasons when each food was accessible.

The Iranian food composition table (FCT)^20^ is incomplete; thus the amounts of energy and nutrient contents were estimated according to the United States Department of Agriculture (USDA) FCT^21^; however, the Iranian FCT was used for some national products (like Kashk) that are not listed in the USDA FCT. Dietary intakes of vitamins E, C, and A and zinc were calculated and considered in grams per week. Vitamin A consumption was considered as taking retinol and its esterified form.

### Physical activity

Physical activity level was assessed using the Persian translation of the modifiable activity questionnaire (MAQ) with high reliability and moderate validity^22^. The time and frequency of the light, moderate, high, and very high intensity activity were obtained according to the list of common activities of daily living over the past year and results were transformed into metabolic equivalent- hours/week (Met/h/week)^23^.

### Blood pressure and anthropometric measurements

Systolic and diastolic blood pressures (SBP and DBP) were measured twice (with a 30 s interval) in a sitting position after 15 min of rest^15,16^.

The body weight was measured to the nearest 100 g, using a digital scale (Seca 707) while the subjects were minimally clothed and without shoes. Height was measured to the nearest 0.5 cm by a tape measure in a standing position without shoes and with shoulders in normal alignment. Waist circumference (WC) was measured with a non-flexible tape meter without any pressure to the body surface at the level of the umbilicus^24^ and was taken at the end of a normal expiration over light clothing. Measurements were recorded to the nearest 0.1 cm.

### Laboratory assays

Blood samples were drawn into vacutainer tubes between 7:00 and 9:00 a.m., after a 12–14-h overnight fast in a sitting position and centrifuged within 30 to 45 min of the collection. All biochemical analyses were performed using a Selectra 2 auto-analyzer at the TLGS research laboratory on the blood collection day. Fasting blood glucose (FBG) concentration was measured on the blood collection day using the colorimetric glucose oxidase procedure. The standard 2-h post-challenge blood glucose test was performed by oral administration of 82.5 g of glucose monohydrate solution (or 75 g of anhydrous glucose) for those who did not take glucose-lowering drugs^15,16^.

High-density lipoprotein cholesterol (HDL-C) concentration was assessed after precipitation of the apolipoprotein B-containing lipoproteins by phosphotungstic acid. Total cholesterol (TC) and triglyceride (TG) were measured using the enzymatic colorimetric method. For TC assay, cholesteryl ester hydrolase was used to convert cholesteryl ester to cholesterol, which was then oxidized by cholesterol oxidase to cholesterol-4-en-3-one and H2O2. TG was broken down to glycerol and free- fatty acids using lipoprotein lipase and glycerol was then phosphorylated to glycerol phosphate by glycerol kinase. Glycerol phosphate was then converted to dihydroxyacetone phosphate and H2O2 by glycerol phosphate oxidase. The Friedewald equation (LDL-C = TC − HDL-C − TG/5) was used to calculate LDL-C concentration in samples containing TG^25^.

### Definitions

Details of the collection of CVD outcome data have been described elsewhere. Coronary heart disease (CHD) events included definite myocardial infarction (diagnostic electrocardiographic [ECG] results and biomarkers), probable myocardial infarction (positive ECG findings plus cardiac symptoms or signs plus missing biomarkers or positive ECG findings plus equivocal biomarkers), proven CHD by angiography, unstable angina pectoris (new cardiac symptoms or changing symptom patterns and positive ECG findings with normal biomarkers), and CHD mortality. CVD was defined as stroke (a new neurological deficit that lasted more than 24 h), CHD events, or CVD death (a fatal stroke or fatal CHD)^26^.

Sex-specific multivariable risk functions (“general CVD” algorithms) were derived that incorporated the age, TC, HDL-C, SBP, treatment for hypertension, smoking, and type 2 diabetes status^27^. Hypertension was defined as SBP ≥ 140, DBP ≥ 90 mmHg, or receiving antihypertensive drug^28^.

### Statistical analyses

Statistical analyses were carried out using the Statistical Package for Social Sciences (version 21.0; SPSS). A two-tailed *P* value < 0.05 was used to determine statistical significance. We used a Chi-square test and Student’s t-test for qualitative and quantitative variables to compare the results obtained from the male and female subjects. For non-normal nutritional and biochemical variables (TG concentration), log-transformed values were used for statistical analysis. Antioxidant intakes with respect to quartiles of food group intakes were assessed using ANOVA test. P for trend was obtained by the linear regression analysis using the median of each quartile as a continuous variable for each food group.

The hazard ratio (HR) and 95% confidence interval (CI) of CVD incidence were assessed using multivariable Cox proportional hazard regression models. Person-years of the follow-up were calculated for each individual from the date inclusion in the study and the exact date of CVD diagnosis, death, or end of the follow-up, whichever came first. Survival time for censored individuals was calculated as the interval between the first and the last observation dates.

The event date was considered as the exact date of CVD events, and survival time was computed as the time between baseline examination and the event date (for event cases) or the last follow-up (for censored cases). Participants were censored due to loss to follow-up, death from a cause other than CVD, or the end of the study without the event appearing.

Rates of CVD-free survival in participants across quartiles of vitamins E, C, and A and zinc intakes were compared using a Kaplan–Meier analysis and the log-rank test method was used for significance test.

The incidence of CVD during the follow-up period was considered as dichotomous variables (yes/no) in the models. The dietary intakes of vitamins E, C, and A, and zinc were adjusted for energy intake using gram per 1000 kcal for each nutrient. Energy adjusted of vitamins E, C, and A, and zinc intakes were categorized into quartiles, given the first quartile as the reference. The median of each quartile was used as a continuous variable to assess the overall trends of HRs across quartiles of dietary intakes of vitamins E, C, and A and zinc in the Cox proportional hazard regression models. Trend test was applied for evaluating dose–response effects in association studies. The proportional hazard assumption of the multivariable Cox models was assessed using Schoenfeld’s global test of residuals.

The confounders were selected based on previous studies and included in the univariable Cox regression model. A two-tailed *P* value < 0.20 was used for inclusion in the model^29^. The Cox regression models were adjusted for age, sex, CVD risk score (continuous), family history of CVD, physical activity (continuous), dietary intakes of total energy, energy-adjusted fiber (g/1000 kcal) and total fat (percentage of energy).

## Results

The mean age of subjects at baseline was 47.0 ± 11.6 and 45.6 ± 10.5 years in men and women, respectively. Table 1 represents the baseline characteristics of men and women. Men were older, had worse smoking habits, greater levels of physical activity, lower body mass index, and higher WC than women. In addition, men had higher values of SBP, DBP, total cholesterol, TG/HDL-ratio, and FBG except for 2 h- plasma glucose. Men had greater amounts of energy and carbohydrate intakes than women. Intakes of total fat, saturated fatty acid, monounsaturated fatty acid, polyunsaturated fatty acid, fiber, and vitamins A and C were higher in women than in men.

**Table 1:** Baseline characteristics of adult participants of the Tehran Lipid and Glucose Study.

	Total sample	Men	Women	*P*
	N = 5102	N = 2253	N = 2849	
Baseline age (years)	46.2 ± 11.1*	47.0 ± 11.6	45.6 ± 10.5	< 0.001
Current smokers (%)	19.1	34.8	6.7	< 0.001
Physical activity (MET/min/week)	524 ± 793	588 ± 844	473 ± 687	< 0.001
BMI (Kg/m^2^)	28.1 ± 4.5	27.1 ± 4.0	28.8 ± 4.8	< 0.001
Waist circumference (cm)	93.8 ± 11.0	96.5 ± 10.3	91.8 ± 11.4	< 0.001
SBP (mmHg)	114 ± 16.7	118 ± 15.9	112 ± 17.1	< 0.001
DBP (mmHg)	76.3 ± 10.2	78.7 ± 10.3	74.4 ± 10.2	< 0.001
Total cholesterol (mg/dl)	219 ± 121	234 ± 138	207 ± 117	< 0.001
LDL (mg/dl)	118 ± 31	118 ± 31	118 ± 32	0.51
TG/HDL-ratio	3.8 ± 3.0	4.6 ± 3.7	3.1 ± 2.4	< 0.001
FPG (mg/dl)	98.1 ± 26.6	99.3 ± 26.2	97.2 ± 27.0	0.007
2 h- plasma glucose (mg/dl)	108 ± 43.1	106 ± 44.5	110 ± 40.3	0.004
Energy intake (kcal/day)	2314 ± 714	2416 ± 728	2230 ± 695	< 0.001
Carbohydrate (% of energy)	58.5 ± 8.3	59.9 ± 6.4	57.5 ± 9.5	< 0.001
Protein (% of energy)	14.6 ± 5.2	14.4 ± 2.7	14.7 ± 8.1	0.19
Total fat (% of energy)	30.4 ± 10.3	28.7 ± 6.0	31.8 ± 17.6	< 0.001
SFA (% of energy)	10.1 ± 11.1	9.5 ± 2.7	10.6 ± 16.9	0.003
MUFA (% of energy)	10.4 ± 11.3	9.7 ± 2.6	10.9 ± 16.9	< 0.001
PUFA (% of energy)	6.3 ± 12.1	5.8 ± 1.9	6.7 ± 16.9	< 0.001
Fiber (g/1000 kcal)	9.7 ± 3.3	9.0 ± 2.8	10.2 ± 3.8	< 0.001
Vitamin E (mg/day)	12.1 ± 5.3	11.2 ± 5.0	12.8 ± 44.5	0.08
Vitamin C (mg/day)	170 ± 123	160 ± 108	178 ± 130	< 0.001
Vitamin A (µg/day)	600 ± 371	565 ± 350	629 ± 399	< 0.001
Zinc (mg/day)	13.7 ± 26.3	14.0 ± 12.4	13.4 ± 45.1	0.58

MET, metabolic equivalent; BMI, body mass index; SBP, systolic blood pressure; DBP, diastolic blood pressure; MUFA, mono-unsaturated fatty acids; PUFA, poly-unsaturated fatty acids; SFA, saturated fat.

*Values are mean ± SD unless otherwise listed.

*P* values derived through Student’s t-test and chi-square test for quantitative and qualitative variables, respectively.

The association between dietary intakes of vitamins E, C, and A and zinc across quartiles of food groups are presented in Table 2. Higher consumption of fruit, vegetable, legumes, refined grains, vegetable oils, fish, and poultry was associated with higher intake of vitamin E. Vitamin C intake was positively associated with all food groups, except for refined grains and red meat that showed a negative association with vitamin C intake. There was a positive association between vitamin A and quartiles of all food groups, except for red meat. Compared with those in the lower quartiles, zinc intake was significantly higher among individuals in the upper quartiles of fruit, vegetable, legumes, whole grains, refined grains, dairy, fish, and poultry intake.

**Table 2:** Antioxidant intakes with respect to quartiles of food group consumption.

Food groups	Vitamin E (mg)	Vitamin C (mg)	Vitamin A (µg)	Zinc (mg)
**Fruits**				
Q1: 0.70 ± 0.27^b^	9.7 ± 4.7	79.7 ± 44.1	421 ± 256	11.3 ± 8.1
Q2: 1.60 ± 0.27	10.8 ± 10.8	123 ± 36.4	534 ± 320	12.9 ± 10.3
Q3: 2.69 ± 0.38	14.0 ± 66.2	178 ± 89.0	625 ± 351	15.3 ± 66.5
Q4: 5.59 ± 2.55	14.1 ± 5.0	300 ± 137	822 ± 446	15.1 ± 14.3
P for trend^c^	< 0.001	< 0.001	< 0.001	0.01
**Vegetables**				
Q1: 1.89 ± 0.53	9.1 ± 4.5	101 ± 83.4	357 ± 212	10.5 ± 8.7
Q2: 3.28 ± 0.36	11.0 ± 4.5	145 ± 94.4	492 ± 213	12.2 ± 7.9
Q3: 4.57 ± 0.39	12.3 ± 5.3	183 ± 92.5	632 ± 278	13.4 ± 10.3
Q4: 7.66 ± 2.82	16.1 ± 66.2	251 ± 142	920 ± 484	18.5 ± 67.3
P for trend	< 0.001	< 0.001	< 0.001	< 0.001
**Legumes**				
Q1: 0.02 ± 0.01	11.6 ± 5.3	157 ± 126	514 ± 341	11.6 ± 6.7
Q2: 0.07 ± 0.01	11.8 ± 5.6	172 ± 122	598 ± 389	13.0 ± 10.6
Q3: 0.15 ± 0.02	11.5 ± 4.8	167 ± 107	613 ± 360	13.7 ± 12.6
Q4: 0.35 ± 0.18	13.6 ± 8.2	183 ± 132	677 ± 407	16.4 ± 67.0
P for trend	< 0.001	< 0.001	< 0.001	< 0.001
**Whole grains**				
Q1: 0.10 ± 0.07	11.2 ± 5.2	149 ± 118	511 ± 344	11.3 ± 8.3
Q2: 0.44 ± 0.13	11.6 ± 5.5	167 ± 107	599 ± 386	12.5 ± 8.7
Q3: 1.31 ± 0.43	13.5 ± 66.2	175 ± 131	622 ± 385	14.1 ± 13.7
Q4: 5.01 ± 3.79	12.5 ± 4.8	189 ± 123	669 ± 384	16.7 ± 66.9
P for trend	0.25	< 0.001	< 0.001	0.002
**Refined grains**				
Q1: 4.90 ± 1.34	10.6 ± 5.7	173 ± 126	614 ± 432	11.0 ± 8.1
Q2: 7.12 ± 0.72	11.3 ± 5.1	168 ± 111	591 ± 347	12.3 ± 10.9
Q3: 10.0 ± 0.95	12.0 ± 5.0	173 ± 118	607 ± 378	14.0 ± 12.8
Q4: 16.6 ± 5.29	14.6 ± 66.2	166 ± 129	590 ± 356	17.4 ± 66.7
P for trend	0.02	0.007	0.004	< 0.001
**Dairy products**				
Q1: 0.92 ± 0.30	12.3 ± 66.3	137 ± 119	449 ± 325	12.5 ± 67.4
Q2: 1.69 ± 0.18	11.2 ± 4.6	161 ± 108	544 ± 332	12.2 ± 7.8
Q3: 2.35 ± 0.22	11.9 ± 5.0	181 ± 125	624 ± 344	13.6 ± 9.4
Q4: 3.71 ± 1.07	13.1 ± 5.5	201 ± 123	785 ± 326	16.2 ± 11.0
P for trend	0.19	< 0.001	< 0.001	< 0.001
**Red meat**				
Q1: 0.07 ± 0.03	12.0 ± 5.4	181 ± 140	612 ± 404	13.3 ± 9.2
Q2: 0.16 ± 0.02	11.7 ± 4.9	172 ± 120	602 ± 349	13.4 ± 12.0
Q3: 0.27 ± 0.04	13.4 ± 66.2	168 ± 122	595 ± 344	14.3 ± 66.3
Q4: 0.60 ± 0.32	11.5 ± 5.6	159 ± 98.6	593 ± 416	13.8 ± 13.8
P for trend	0.70	< 0.001	0.12	0.32
**Fish and poultry**				
Q1: 0.41 ± 0.15	10.7 ± 4.9	148 ± 116	499 ± 346	11.3 ± 8.3
Q2: 0.84 ± 0.13	11.2 ± 4.9	161 ± 112	554 ± 318	12.5 ± 8.7
Q3: 1.32 ± 0.16	12.1 ± 5.0	174 ± 112	627 ± 386	14.1 ± 13.7
Q4: 2.92 ± 1.87	14.6 ± 66.3	197 ± 137	722 ± 498	16.7 ± 66.9
P for trend	0.001	< 0.001	< 0.001	< 0.001
**Sugar-sweetened soft drinks (ml/d)**				
Q1: 0.76 ± 0.95	13.1 ± 66.1	170 ± 131	595 ± 382	14.2 ± 66.4
Q2: 8.29 ± 2.23	11.1 ± 4.6	159 ± 117	560 ± 376	12.7 ± 11.2
Q3: 24.3 ± 7.59	11.7 ± 4.8	174 ± 122	622 ± 380	14.1 ± 14.4
Q4: 109 ± 101	12.6 ± 5.7	176 ± 113	626 ± 377	13.8 ± 7.6
P for trend	0.55	< 0.001	< 0.001	0.68
**Vegetable oils**				
Q1: 2.13 ± 1.25	8.4 ± 3.5	138 ± 98	497 ± 311	11.7 ± 9.8
Q2: 6.27 ± 0.73	10.6 ± 3.5	163 ± 116	563 ± 341	13.0 ± 13.2
Q3: 9.26 ± 1.31	13.7 ± 65.9	184 ± 141	641 ± 392	15.5 ± 66.5
Q4: 18.62 ± 8.98	16.1 ± 6.4	196 ± 118	703 ± 434	14.5 ± 9.3
P for trend	0.04	0.07	0.04	0.30
**Nuts**				
Q1: 0.02 ± 0.01	11.2 ± 5.1	145 ± 109	537 ± 379	12.5 ± 108
Q2: 0.08 ± 0.01	13.6 ± 66.2	169 ± 135	596 ± 364	15.0 ± 66.7
Q3: 0.17 ± 0.03	11.7 ± 11.7	177 ± 113	621 ± 373	13.4 ± 11.7
Q4: 0.55 ± 0.42	12.0 ± 5.6	188 ± 188	648 ± 393	13.8 ± 10.7
P for trend	0.96	< 0.001	< 0.001	0.33
**Tea and coffee (ml/d)**				
Q1: 200 ± 88.1	12.7 ± 66.4	156 ± 123	549 ± 359	14.5 ± 67.2
Q2: 379 ± 112	11.5 ± 4.8	169 ± 118	612 ± 398	13.1 ± 11.2
Q3: 679 ± 101	12.0 ± 4.6	175 ± 121	613 ± 342	13.5 ± 11.2
Q4: 1342 ± 741	12.5 ± 6.3	179 ± 121	628 ± 383	13.6 ± 7.8
P for trend	0.50	< 0.001	0.001	0.97

^a^Values are serving/day, with the exception of sugar-sweetened soft drinks, Tea and coffee.

^b^Data are mean ± SD using ANOVA test.

^c^P for trend were obtained by the linear regression analysis using the median of each quartile as a continuous variable for each food group.

**Figure 2:**
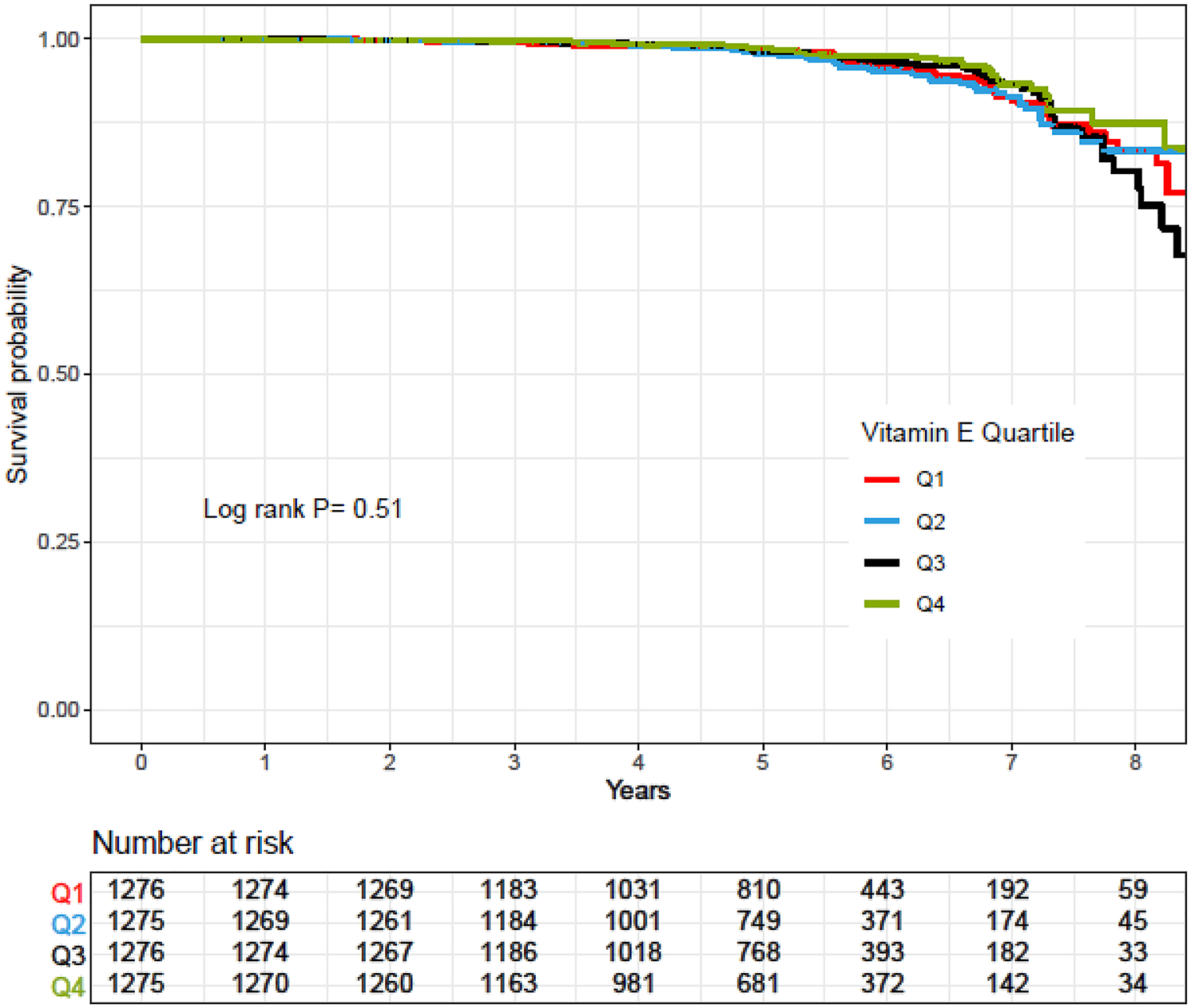
Kaplan–Meier estimates of CVD-free survival according to quartiles of vitamin E intake. *P* values by log-rank test and number of CVD outcomes recorded in the groups categorized by vitamin E intake.

**Figure 3:**
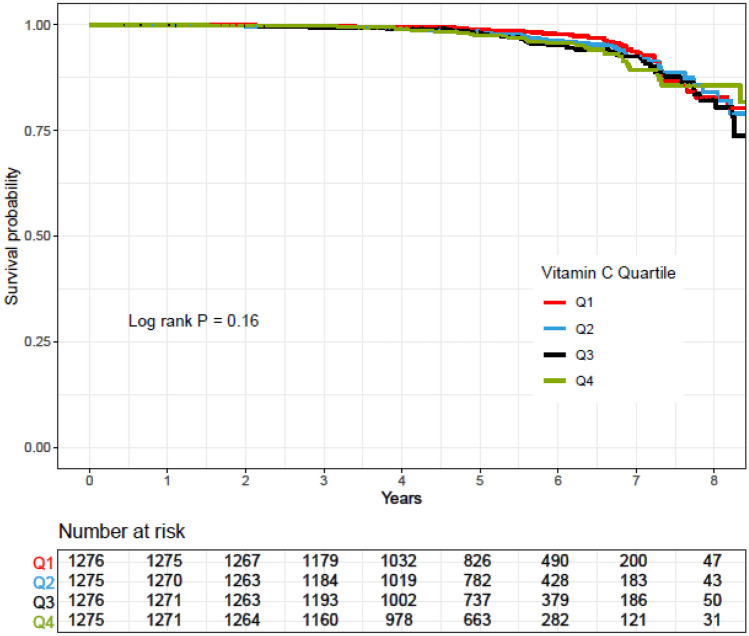
Kaplan–Meier estimates of CVD-free survival according to quartiles of vitamin C intake. *P* values by log-rank test and number of CVD outcomes recorded in the groups categorized by vitamin C intake.

**Figure 4:**
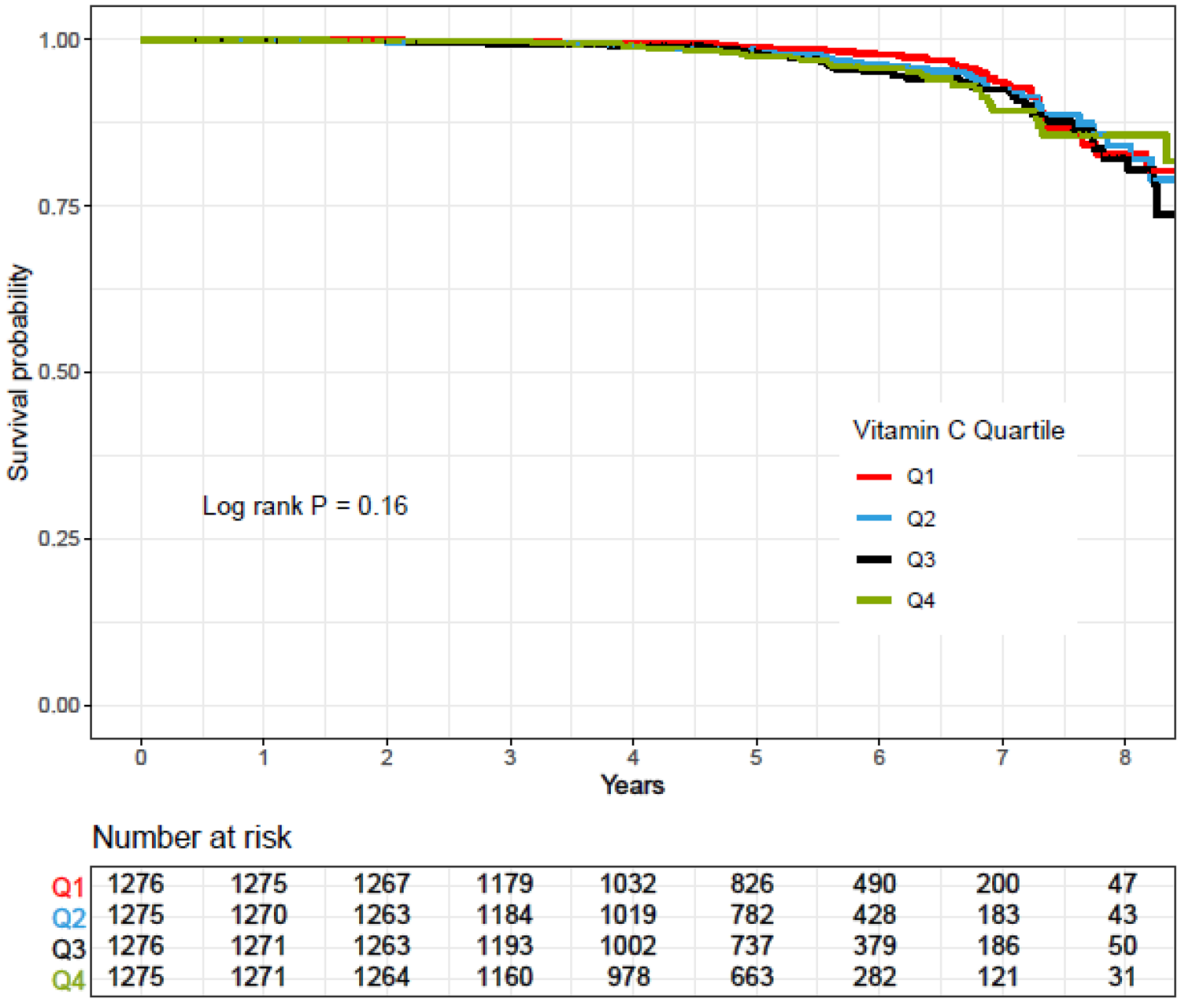
Kaplan–Meier estimates of CVD-free survival according to quartiles of vitamin A intake. *P* values by log-rank test and number of CVD outcomes recorded in the groups categorized by vitamin A intake.

**Figure 5:**
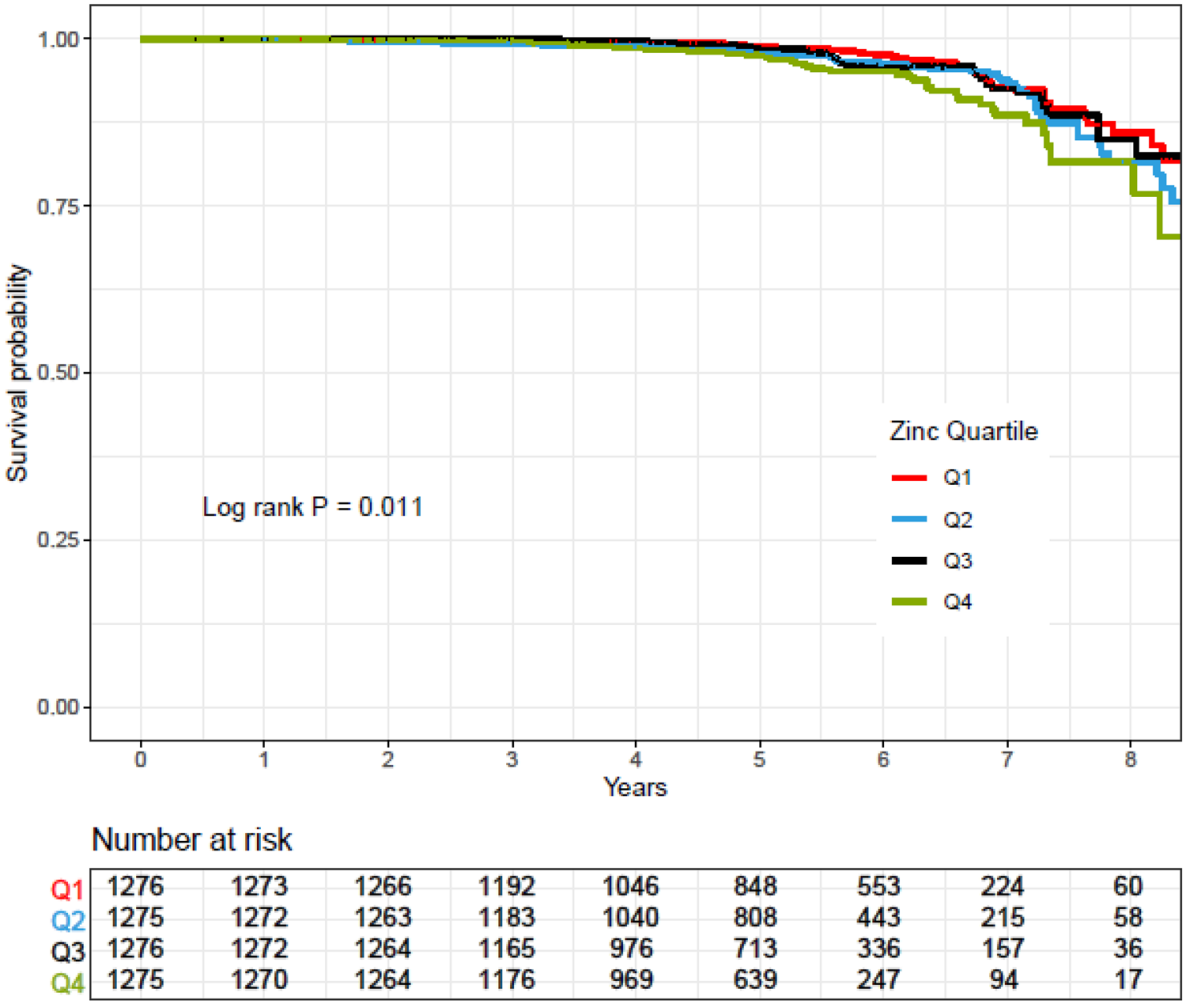
Kaplan–Meier estimates of CVD-free survival according to quartiles of zinc intake. *P* values by log-rank test and number of CVD outcomes recorded in the groups categorized by zinc intake.

Kaplan–Meier survival curves for CVD according to quartiles of vitamins A, C, and E and zinc intakes during follow-up periods are shown in Figs. 2, 3, 4 and 5. Significant differences were found in the risk of CVD between quartiles of zinc intake.

HRs (95% CI) of CVD for quartiles of dietary antioxidants (vitamins A, E, and C, and zinc) intakes are presented in Table 3. After adjustment for potential confounders, the risk of CVD decreased from quartile 1 to quartile 4 for vitamin E intake [HR 1.00, 0.91 (0.62–1.64), 0.77 (0.51–1.18), and 0.57 (0.34–0.97); *P*_trend_ = 0.03]. The association between the risk of CVD and quartiles of vitamins A and C, and zinc intake were not statistically significant.

**Table 3:** Hazard Ratios (HR) and 95% CIs for cardiovascular diseases according to quartiles of dietary antioxidants intake.

Variables	Quartiles of intake				
	Q1	Q2	Q3	Q4	*P*_trend_^a^
**Vitamin E intake**					
Median, mg/d	6.8	9.5	12.4	16.8	
Cases, n	61	56	51	38	
Person-years, n	6711	6530	6593	6423	
Follow-up, y	5.3	5.2	5.2	5.1	
Incidence, %	9.0	8.5	7.7	5.9	
Crude	1.00 ref	0.94 (0.65–1.35)	0.85 (0.58–1.23)	0.65 (0.43–0.98)	0.03
Model adjusted^b^	1.00 ref	0.91 (0.62–1.64)	0.77 (0.51–1.18)	0.57 (0.34–0.97)	0.03
**Vitamin C intake**					
Median, mg/d	64.7	114.2	173.3	291.6	
Cases, n	55	52	63	46	
Person-years, n	6844	6643	6515	6256	
Follow-up, y	5.5	5.3	5.1	5.0	
Incidence, %	6.5	7.8	9.6	7.3	
Crude	1.00 ref	1.19 (0.80–1.77)	1.47 (1.00–2.16)	1.13 (0.74–1.70)	0.58
Model adjusted ^b^	1.00 ref	1.04 (0.68–1.59)	1.19 (0.76–1.85)	0.84 (0.48–1.48)	0.44
**Vitamin A intake**					
Median, µg/d	267	428	611	964	
Cases, n	53	60	41	52	
Person-years, n	7020	6020	6472	6145	
Follow-up, y	5.7	5.3	5.1	5.0	
Incidence, %	7.5	9.0	6.3	8.4	
Crude	1.00 ref	1.21 (0.83–1.75)	0.84 (0.56–1.27)	1.13 (0.77–1.67)	0.81
Model adjusted^b^	1.00 ref	1.16 (0.78–1.70)	0.82 (0.52–1.28)	1.00 (0.61–1.63)	0.74
**Zinc**					
Median intake (mg/d)	7.6	10.5	13.2	17.8	
Cases, n	51	61	41	53	
Person-years, n	6959	6728	6409	6161	
Follow-up, y	5.6	5.3	5.1	5.0	
Incidence, %	7.3	9.0	6.3	8.6	
Crude	1.00 ref	1.24 (0.85–1.80)	0.88 (0.58–1.33)	1.16 (0.81–1.76)	0.62
Model adjusted^b^	1.00 ref	1.20 (0.80–1.81)	0.81 (0.49–1.35)	1.09 (0.58–2.05)	0.97

^a^Test for trend based on ordinal variable containing median value for each quartile.

^b^Adjusted for age, sex, CVD risk score, family history of CVD, physical activity, total energy intake, fiber and total fat intakes.

## Discussion

The current investigation was a prospective cohort study, evaluating the association between dietary antioxidant (vitamins A, E, and C and zinc) intakes and risk of CVD. Our results suggested that a higher intake of vitamin E was inversely associated with CVD incidence.

Previous studies have found an association between dietary vitamin E intake and decreased risk of CVD in observational epidemiologic studies^28,30–32^. Several biological functions of vitamin E are due to its antioxidant properties to inhibit the oxidation of LDL-C and to scavenge lipid radicals^33^. In contrast, several large randomized controlled trials have failed to corroborate the benefits of vitamin E in CVD prevention^34^, which can be due to several factors, such as the time of intervention, gene polymorphisms, or inherent confounding, and pathophysiological conditions in study populations. In addition, an intervention study suggested that in addition to prescribed medicine, supplemental doses of vitamin E should be given to ameliorate therapeutic strategies^35^. However, vitamin E supplements have not been recommended by the American heart association to prevent CVD due to the lack of approved results; however, it recommends eating foods rich in antioxidant vitamins, especially fruit and vegetable^36^.

In our study, vitamin E was available in several food groups, which may be due to the connection between food groups, such as consuming oil (olive oil and mayonnaise) and vegetable that are mixed in salads or using oil for cooking. Also, no association was found between nut intake and vitamin E, which may be due to the small quantities of nut consumption in our population.

Anti-CVD properties of vitamin C have not yet been fully confirmed. No significant association was found between total vitamin C intake (estimated by summing the vitamin C contribution of food items and supplements) and CVD in a cohort of Spanish university graduates. Means of fiber-adjusted dietary vitamin C intake (mg/day) across tertiles were 184, 266, and 387, respectively. Therefore, it is concluded that the absence of significant results can be due to the low variability in the exposure. However, vitamin C is a single nutrient and may not represent the synergistic effect of the whole dietary pattern^37^. In contrast to our results, observational studies on the relationship between vitamin C and CVD risk have demonstrated an inverse association between vitamin C and CVD outcomes, especially heart failure^7^ and hypertension^8^. The contradiction in results can be due to differences in the definition of CVD. Vitamin C increases the nitric oxide bioactivity of the endothelium, which causes a decrease in blood pressure^38^. Moreover, vitamin C reduces monocyte adhesion and inhibits LDL oxidation^39^, which plays an important role in decreasing the risk of atherosclerosis. In addition, vitamin C keeps atheromatous plaques stable by preventing vascular smooth muscle cell apoptosis^40^.

Consistent with our results, in a meta-analysis^41^, neither dietary nor supplemental vitamin A was associated with CVD risk. A large prospective study^10^ indicated that among 4117 patients with stable angina pectoris in the upper tertile of serum vitamin A concentration, serum apolipoprotein B (a predictor of CVD) was associated with the CVD risk. However, dietary intake of vitamin A did not correlate with serum concentration, which seems that another mechanism, other than vitamin A intake, regulates the serum vitamin A concentration^42^. Accordingly, in the current study, no association was observed between dietary vitamin A and CVD risk.

In the present study, no significant difference was found between dietary zinc intake and CVD incidence, which is consistent with a systematic review of prospective cohort studies on the association between dietary zinc intake or serum zinc levels and the incidence of CVD^43^. In contrast with our result, higher dietary zinc intake (estimated by summing the zinc contribution of food items) was associated with a greater incidence of CVD in a large longitudinal study on Australian women. Therefore, more investigations are needed to investigate the association between zinc intake from meat and other major sources because dietary guidelines recommend reducing red meat intake and encouraging consuming other sources of zinc^44^. Also, more studies are needed to investigate the mechanisms of zinc function on the pathogenesis of CVD to provide dietary zinc recommendations for preventing CVD.

The prospective design of the present study was one of its major strengths that facilitated the estimation of disease incidence without concern about reverse causality between nutrients and outcomes. In this research, the subjects’ reports regarding food consumption were used, whereas multiple assessments of urine or circulating biomarkers of vitamins over time could be a more reliable approach and it was one of the limitations of this study. Considering the observational design of the current research, some confounders (e.g. supplement intake and socioeconomic levels) and comorbidities during follow-up were not considered. Dietary assessment was considered at baseline only, and changes in dietary intakes were not recorded during the follow-up. One of the limitations of this study was the use of an FFQ for collecting dietary data due to recall bias; however, expert dietitians interviewed the participants to reduce this bias. Also, the validity of the FFQ was acceptable among this population.

Moreover, some cases lost the follow-up; for example, those with a high risk of CVD were selectively excluded due to their poor mental or physical health. We did not split the analysis by gender due to the low number of cases and power reduction.

## Conclusion

This study suggested an inverse association between vitamin E intake and the risk of CVD, which emphasized the potentially protective role of its dietary sources in the prevention of CVD events.
